# Prediction of 2-Year Major Adverse Limb Event-Free Survival After Percutaneous Transluminal Angioplasty and Stenting for Lower Limb Atherosclerosis Obliterans: A Machine Learning-Based Study

**DOI:** 10.3389/fcvm.2022.783336

**Published:** 2022-02-09

**Authors:** Tianyue Pan, Xiaolang Jiang, Hao Liu, Yifan Liu, Weiguo Fu, Zhihui Dong

**Affiliations:** ^1^Department of Vascular Surgery, Institute of Vascular Surgery, Zhongshan Hospital, Fudan University, Shanghai, China; ^2^National Clinical Research Center for Interventional Medicine, Shanghai, China

**Keywords:** machine learning, lower limb atherosclerosis obliterans, endovascular therapy, prognosis prediction, major adverse limb events

## Abstract

**Background:**

The current scoring systems could not predict prognosis after endovascular therapy for peripheral artery disease. Machine learning could make predictions for future events by learning a specific pattern from existing data. This study aimed to demonstrate machine learning could make an accurate prediction for 2-year major adverse limb event-free survival (MFS) after percutaneous transluminal angioplasty (PTA) and stenting for lower limb atherosclerosis obliterans (ASO).

**Methods:**

A lower limb ASO cohort of 392 patients who received PTA and stenting was split to the training set and test set by 4:1 in chronological order. Demographic, medical, and imaging data were used to build machine learning models to predict 2-year MFS. The discrimination and calibration of artificial neural network (ANN) and random forest models were compared with the logistic regression model, using the area under the receiver operating curve (ROCAUC) with DeLong test, and the calibration curve with Hosmer–Lemeshow goodness-of-fit test, respectively.

**Results:**

The ANN model (ROCAUC = 0.80, 95% CI: 0.68–0.89) but not the random forest model (ROCAUC = 0.78, 95% CI: 0.66–0.87) significantly outperformed the logistic regression model (ROCAUC = 0.73, 95% CI: 0.60–0.83, *P* = 0.01 and *P* = 0.24). The ANN model the logistic regression model demonstrated good calibration performance (*P* = 0.73 and *P* = 0.28), while the random forest model showed poor calibration (*P* < 0.01). The calibration curve of the ANN model was visually the closest to the perfectly calibrated line.

**Conclusion:**

Machine learning models could accurately predict 2-year MFS after PTA and stenting for lower limb ASO, in which the ANN model had better discrimination and calibration. Machine learning-derived prediction tools might be clinically useful to automatically identify candidates for PTA and stenting.

## Introduction

Peripheral arterial disease (PAD), frequently caused by atherosclerosis, could progress to arterial stenosis or occlusion and lead to chronic or acute limb ischemia. Lower limb arteriosclerosis obliterans (ASO) is the main subtype of PAD, characterized by claudication in the early stage, and rest pain and non-healing wound in the end stage, which is called critical limb ischemia (CLI). As the population ages, the morbidity of ASO is increasing and the incidence rate of CLI was estimated to be 500–1,000 per year per million in a European or North American population (1). Currently, open bypass surgery and endovascular intervention are the main treatment options for ASO-related CLI or severe claudication. With the advantage of minimally invasive and rapid recovery, endovascular intervention is considered the first choice for patients with a high risk of complications (2).

The Rutherford and Fontaine scoring systems were firstly established in the 20th century to assess the severity of limb ischemia before the age of “endovascular.” These scoring systems, though still widely used, could afford limited information about prognosis after revascularization. In 2014, the Society of Vascular Surgery proposed the wound, ischemia, foot infection (WIFI) scoring system to evaluate 1-year limb amputation risk and revascularization benefit (3). The WIFI system is widely accepted and has expanded to predict the prognosis of revascularization (4, 5). The Trans-Atlantic Inter-Society Consensus committee (TASC) and the recent Global Limb anatomic Staging System (GLASS) grading systems, on the other hand, focused on the anatomic characteristics and could suggest option and technical success rate of a certain type of intervention (2). Nowadays, endovascular intervention techniques are quickly developing and are increasingly applied for the treatment of lower limb ASO, even in TASC C and D subgroups. Healthcare providers of PAD have a growing need for an easily interpretable tool that could comprehensively consider factors of demography, limb ischemia, anatomy, and intervention to predict prognosis and further identify candidate patients for endovascular intervention. In clinical practice, percutaneous transluminal angioplasty (PTA) with selective stenting is the main strategy of endovascular intervention iliac, superficial femoral, popliteal and infrapopliteal stenotic, and occlusive lesions. Stenting is generally considered inevitable for those with severe residual stenosis, obvious dissection after balloon angioplasty, and thrombosis. The prognoses after stenting vary between patients and might be influenced by systemic factors, the severity of ischemia, lesion characteristics (like chronic total occlusion and calcification), and stent parameters (diameter and total length). Unfortunately, the current scoring systems could hardly afford a comprehensive consideration of the overall effect of these factors. A practitioner could also hardly make an accurate prediction of prognosis after stenting even though he or she had a wealth of experience in vascular surgery. Here, we aimed to use machine learning to automatically create an algorithm to calculate the probability of prognosis after receiving lower limb PTA with stenting. This might help identify candidates for PTA and stenting, and discriminate patients unsuitable for endovascular intervention and avoid inappropriate stent placement.

Machine learning technology could make predictions for future events by learning a specific relationship pattern between multiple variables from existing data. It has been demonstrated that machine learning could create algorithms for accurate prediction for PAD or cardiovascular events based on clinical data of multiple dimensions (6, 7). In this study, we decided to use two different modern supervised learning algorithms to train and validate models for prediction of 2-year major adverse limb event (MALE)-free survival (MFS) after PTA with stenting for lower limb ASO. One of the methods is called Random forest, a popular ensemble learning method that could significantly reinforce the predictive ability of classic decision tree-based algorithm. Another method is called artificial neural network (ANN), which could train an optimized prediction mode by automatically learning weights of interconnecting neurons. We compared these two models with the standard multivariate logistic regression model to find out whether machine learning could bring a significant improvement of predictive ability for prognosis.

## Materials and Methods

### Study Population

This study was performed based on a retrospectively enrolled cohort of patients who were diagnosed with lower limb ASO and received primary successful treatment of PTA with stenting from January 1st, 2016 to December 30th, 2017 in our center. The patients finally included in the study met the following criteria: (1) diagnosed as lower limb ischemia due to arterial stenosis or occlusion and the etiology was atherosclerosis obliterans; (2) Rutherford's grade of 2–6; (3) receiving PTA and stenting successfully for the first time; (4) target lesion for stenting located in common iliac artery, external iliac artery, superficial femoral artery, or popliteal artery; and (5) complete baseline data and known status of survival and lower limb at 2 years postsurgery. Exclusion criteria included: (1) any serious health events which might mislead the assessment of lower limb function including, but not limited to heart failure, symptomatic cerebral apoplexy, and lower limb fracture before admission; (2) lower limb ischemia due to other etiologies including, but not limited to arterial embolism, angiitis, and arterial aneurysm before admission; (3) diagnosed with malignancy at baseline; and (4) any surgery or endovascular intervention for the target lower limb artery performed before.

This study was approved by the Ethical Committee of Zhongshan Hospital, Fudan University, China. The informed consent was waived due to the retrospective nature of this study. It was performed in agreement with the ethical principles of the Declaration of Helsinki.

### Baseline Information and Endpoint

Data collected from the medical records involved demographic data, risk factors of peripheral artery disease, cardiovascular and cerebrovascular disease history, blood examination, namely, creatinine, fibrinogen, neutrophil-lymphocyte ratio, and platelet-lymphocyte ratio. The features of the treated limb included ischemia status, ankle-brachial index (ABI), and targeted lesion properties, namely, location, type, length, calcification, and infrapopliteal runoff based on computed tomography angiography (CTA) and intraoperative angiography. The peripheral arterial calcium scoring system (PACSS) was applied to access calcification (8). The endpoint was MALE or death in 2 years after intervention. Determination of MALE should meet one of the following criteria: (1) re-intervention for the target lesion, (2) above-ankle amputation (major amputation) in the treated limb, and (3) recurrence of claudication, rest pain or tissue loss in the treated limb with ABI decreased >0.2. Two groups of clinical staff collected baseline information and endpoints, respectively, and independently.

### Treatment Protocol

Ankle-brachial index (ABI) test and lower limb CTA were performed regularly to determine the target lesion location and the access route. During the endovascular therapy, an arteriogram was performed to evaluate the target lesion type and calcification grade. Guidewire and catheter were used to get through the lesion using antegrade or/and retrograde approach. Once the true lumen of the distal runoff was confirmed, balloon dilation and stenting were performed to reconstruct the target lesion with or without debulking. The indication of selective stenting included residual stenosis after balloon dilation >30%, obvious or flow-limiting dissection, and thrombosis. The length of the target lesion for stenting was measured. Arteriogram was then performed to confirm the vascular patency and ensure no thrombosis, distal embolism or flow-limited dissection existed. It should be noted the patients who received only balloon dilation or debulking without stenting were not included in this study. After the intervention, dual-antiplatelet was regularly used, and anticoagulation was selectively used in patients with thrombosis lesions. The compliance of dual-antiplatelet was recorded for each patient during the follow-up, and poor compliance was defined as forgetting to take medicine >2 in 2 weeks or unwillingness to insist on taking medicine.

### Machine Learning

**Figure 1:**
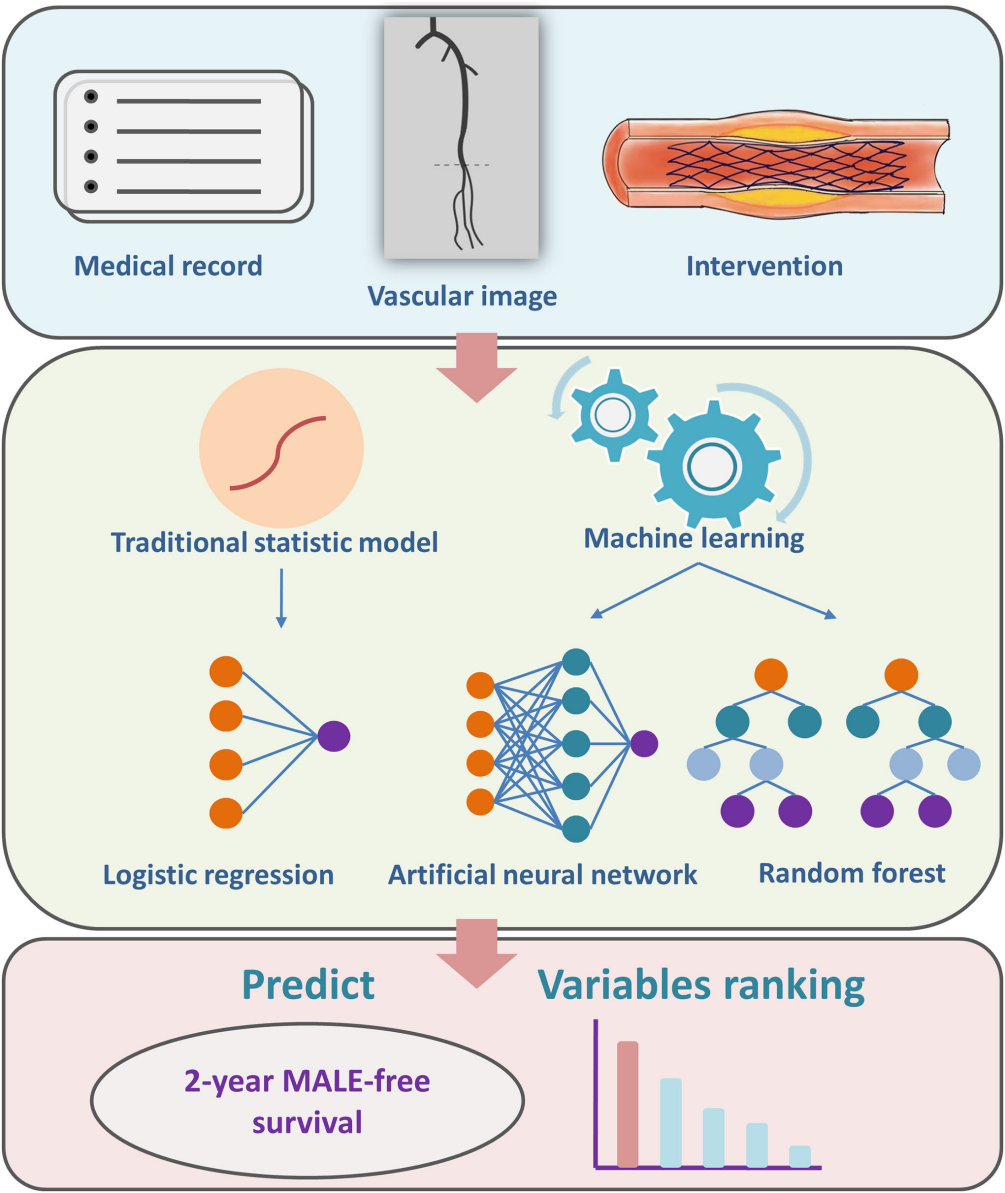
A brief summary of study design and machine learning model development. MALE, major adverse limb event.

Machine learning refers to computational methods that could make explanations and predictions for future events by learning a specific pattern from existing data. In this study, two popular supervised machine learning algorithm methods called random forest and ANN were used to train models to predict 2-year MFS after PTA and stenting for lower limb ASO. A traditional multivariate logistic regression was used as a standard method. The development of machine learning models was briefly shown in Figure 1.

The whole cohort that met the inclusion and exclusion criteria was split into the training set and the test set by 4:1 in chronological order. The machine learning models were trained and cross-validated validated (for the ANN model) on the training set, and the test set was used to evaluate the discrimination and calibration of these trained models.

### Model Training

The variables for model training were screened through the univariate logistic regression analysis in the training set. Those with an odds ratio *P*-value <0.1 were considered as candidate variables for the training of the machine learning models. Any variable which was considered clinically important to prognosis was also chosen for model training even though the odds ratio *P*-value was >0.1.

The ANN model used in this study was also called multilayer perceptrons. The structure of the model consists of an input layer that receives and transfers variable values, one or more hidden layers of neurons processing the signals from the previous layers through weights in a non-linear pattern, and an output layer receiving signals from the hidden layers to calculate a probability value. The error between the probability value and the true value calculated by the loss function is backpropagated through the neural network and update weights of neurons in the hidden layers during each epoch. Finally, the optimal ANN model was trained. In our study, the loss function was binary cross-entropy and the optimizer was Adam. Besides, L2 regularization and dropout methods were added to reduce overfitting. A ten-fold cross-validation was applied in the pretraining phase of the model to monitor the overfitting and help to determine the proper number of the epoch, layer, and neuron for the final training. The final ANN model was trained using the whole training set with optimized parameters.

Random forest is a popular ensemble learning model introduced by Leo Breiman and Adele Cutler (9). This model adds “random” into the bagging method to reinforce the classification and generalization ability of the decision tree-based classifier algorithm. The random forest grows many decision tree classifiers to which samples and variables are randomly assorted and there is no pruning during the growth. Each tree votes for a classification, and the forest finally chooses the classification having the most votes. Furthermore, the random forest model could give importance to each variable after model training. The importance value of each variable is calculated according to error comparison between specific variable values in “out of bag” data before and after random rearrangement. It is noteworthy that cross-validation is not needed in the training phase due to the nature of the random forest algorithm. In our study, the parameters were set as following: number of trees (n_estimators) = 100, criterion = gini, max feature = square root of variable number, max depth = not limited, min samples split = 2, min sample leaf = 1, and max-leaf nodes = not limited.

The traditional multivariate logistic regression model was set as a standard method to test the performance of the ANN and random forest model. It was trained on the training set using the same variables as the other models and then applied for prediction in the test set.

### Model Testing

The discrimination of the models was assessed using the area under the receiver operating curve (ROCAUC) as metrics and the ROCAUC was compared using the DeLong test. Sensitivity and specificity at a cutoff value of 0.5 were also used as metrics. The calibration was visually assessed by depicting the curve of the predicted and the observed probabilities and assessed for significance using Hosmer–Lemeshow test.

The algorithms of training, validation, and test of machine learning models were written using Python language (Version 3.6.8, https://www.python.org) with Keras frame (Tensorflow backend, Google Incorporation, USA) and the scikit-learn package of machine learning.

### Statistical Analysis

The baseline characteristics between the patients with and without endpoints in the training set were compared. Continuous variables were presented as means ± SDs or medians ± interquartile ranges (IQRs) according to the data distribution. The independent Student's *t*-test or Mann–Whitney *U* test was used to analyze the significance of the differences. Categorical variables were presented as numbers with percentages, and Pearson's chi-squared test or Fisher's test was used to analyzing the significance of the differences. A two-tailed *P*-value < 0.05 was considered as statistically significant. The statistical analyses were performed with SPSS (version 19.0, Chicago, Illinois, USA) and R (version 4.1.0, https://www.r-project.org).

## Results

### Baseline Information

**Figure 2:**
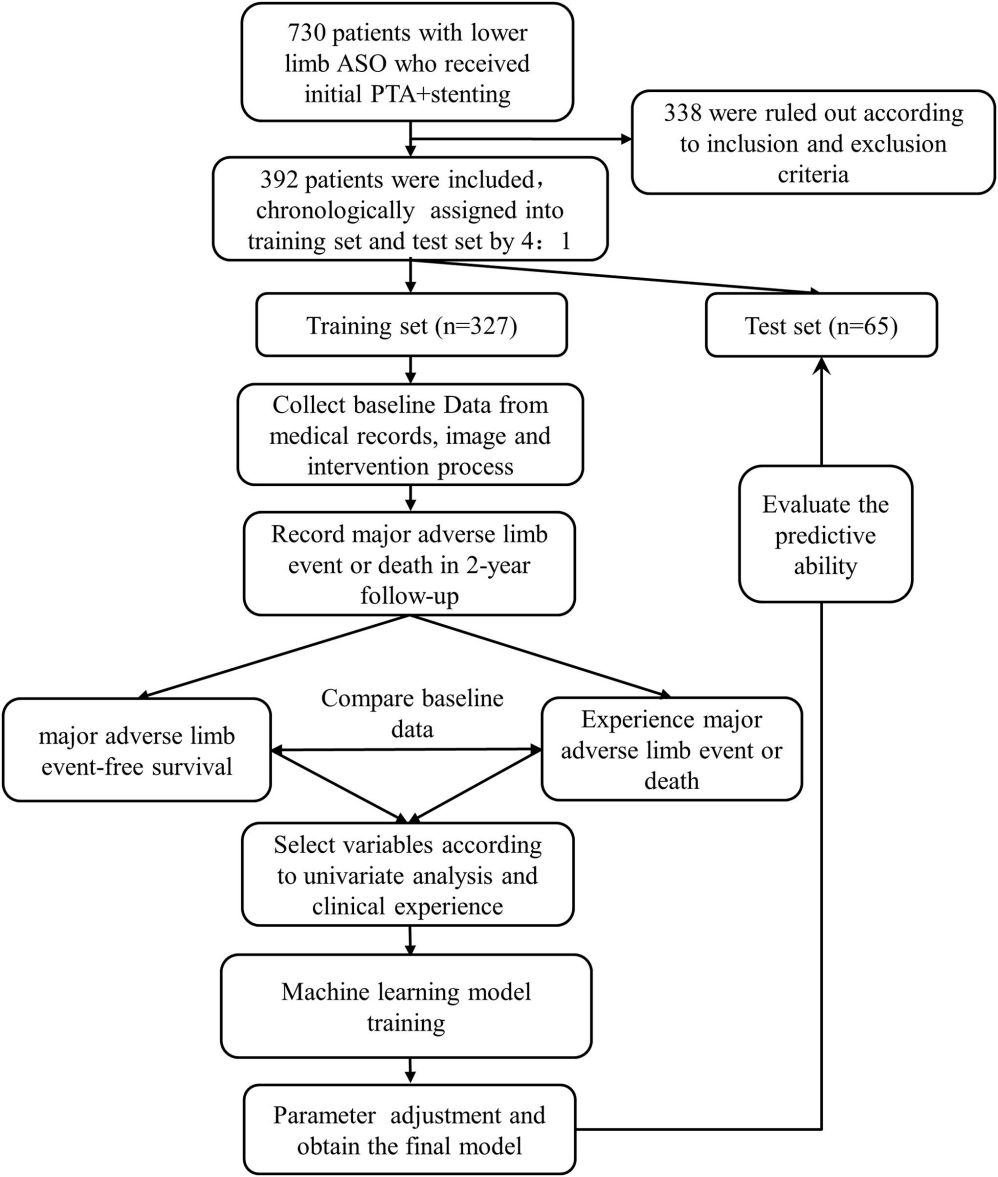
Study flow diagram. PTA, percutaneous transluminal angioplasty.

A total of 730 patients were screened and 392 patients were finally included in this study according to the inclusion and exclusion criteria (Figure 2). A total of 327 patients (80%) were allocated to the training set, of which 271 were male (82.9 %) and the average age was 71.6 ± 9.7 years. In the training set, the 2-year MFS rate was 59.6%, while 104 patients experienced MALE and 28 were dead. A comparison of the baseline characteristics between the two groups of patients with and without the endpoint in the training set was summarized in Table 1.

**Table 1:** Baseline characteristics of the training set.

**Baseline characteristics**	**Total, *n* = 327**	**MOD, *n* = 132**	**MFS, *n* = 195**	***P*-value**
**Age (years) (mean** **±SD)**	71.6 ± 9.7	73.4 ± 9.5	70.4 ± 9.6	0.005
≤ 80, *n* (%)	260 (79.5%)	96 (72.7%)	164 (84.1%)	0.012
>80, *n* (%)	67 (20.5%)	36 (27.3%)	31 (15.9%)	0.012
**Gender (male)**, ***n*** **(%)**	271 (82.9%)	104 (78.8%)	167 (85.6%)	0.107
**Risk factors**				
Hypertension, *n* (%)	244 (74.6%)	93 (70.5%)	151 (77.4%)	0.155
Diabetes mellitus, *n* (%)	132 (40.4%)	58 (43.9%)	121 (37.9%)	0.279
Hyperlipidemia, *n* (%)	91 (27.8%)	39 (29.5%)	52 (26.7%)	0.569
Myocardial infarction, *n* (%)	77 (23.5%)	29 (22.0%)	48 (24.6%)	0.580
Stroke, *n* (%)	55 (16.8%)	22 (16.7%)	33 (16.9%)	0.952
Current smoker, *n* (%)	77 (23.5%)	34 (25.8%)	43 (22.1%)	0.438
**Blood examination**				
Fibrinogen (mg/dl) [median (IQR)]	309.0 (263.5–387.0)	312.0(261.0–404.0)	307.0 (265.0–379.5)	0.439
Creatinine (μmol/L) [median (IQR)]	86.0 (72.0–107.5)	86.0 (71.0–110.0)	85.0 (73.0–104.0)	0.813
NLR [median (IQR)]	3.8 (2.5–5.6)	3.8 (2.3–5.6)	3.8 (2.5–5.5)	0.881
PLR [median (IQR)]	135.1 (100.6–201.1)	127.4 (92.9–204.9)	136.3 (103.6–190.0)	0.537
**Target limb**				
Rest pain or wound	149 (45.6%)	70 (53.0%)	79 (40.5%)	0.026
ABI	0.48 ± 0.20	0.43 ± 0.18	0.52 ± 0.21	<0.001
**Target lesion location**				
Iliac	109 (33.3%)	37 (28.0%)	72(36.9%)	0.078
Femoral-popliteal	181 (55.4%)	83 (62.9%)	98 (50.3%)	0.078
Mixed	37 (11.3%)	12 (9.1%)	25 (12.8%)	0.078
Target lesion length	10.0 (6.0–20.0)	12.5 (8.0–24.5)	8.5 (5.0–16.0)	<0.001
**Target lesion type**				
Chronic total occlusion	226 (69.1%)	98 (74.2%)	128 (65.6%)	0.251
Stenosis	90 (27.5%)	30 (22.7%)	60 (30.8%)	0.251
Thrombosis	11 (3.4%)	4 (3.0%)	7 (3.6%)	0.251
**PACSS**				
0–3	275 (84.1%)	88 (66.7%)	187 (95.9%)	<0.001
4	52 (15.9%)	44 (33.3%)	8 (4.1%)	<0.001
**Infra-popliteal runoff**				
No patent tibial artery	11 (3.4%)	6 (4.5%)	5 (2.6%)	0.008
1 patent tibial artery	84 (25.7%)	43 (32.6%)	41 (21.0%)	0.008
2 patient tibial arteries	84 (25.7%)	38 (28.8%)	46 (23.6%)	0.008
3 patent tibial arteries	148 (45.2%)	45 (34.1%)	103 (52.8%)	0.008
**Intervention**				
Total stent length [median (IQR)]	12.0 (8.0–25.0)	15.0 (10.0–28.0)	12.0 (8.0–23.0)	0.021
Minimal stent diameter	6.3 ± 1.4	6.0 ± 1.4	6.6 ± 1.3	<0.001
Debulking	21 (6.4%)	11 (8.3%)	10 (5.1%)	0.246
Retrograde puncture	70 (21.4%)	31 (23.5%)	39 (20.0%)	0.451
**Medication**				
Dual anti-platelet	327 (100%)	132 (100%)	195 (100%)	1.000
Anti-coagulant	11 (3.4%)	4 (3.0%)	7 (3.6%)	0.783
Statin	99 (30.3%)	37 (28.0%)	62 (31.8%)	0.467
Poor compliance of anti-platelet	80 (24.5%)	36 (27.3%)	44 (22.6%)	0.331

*MOD, major adverse limb event or death; MFS, major adverse limb event-free survival; SD, standard deviation; IQR, interquartile range; ABI, ankle-brachial index; PACSS, peripheral artery calcification scoring system; NLR, neutrophil-lymphocyte ratio; PLR, platelet-lymphocyte ratio*.

### Candidate Variables

The univariate logistic regression analysis identified 8 variables were associated significantly with the endpoint (*P* < 0.05) (Table 2). Those variables included age > 80 years [odds ratio (OR) =1.984, 95% CI: 1.153–3.412, *P* = 0.013], rest pain or wound (OR = 1.658, 95% CI: 1.062–2.589, *P* = 0.026), ABI < 0.4 (OR = 2.599, 95% CI: 1.633–4.137, *P* < 0.001), lesion location (OR = 1.648, 95% CI: 1.007–2.697), patent infrapopliteal arteries < 3 (OR = 2.164, 95% CI: 1.371–3.417, *P* = 0.001), target lesion length > 20 cm (OR = 3.143, 95% CI: 1.857–5.320, *P* < 0.001), PACSS = 4 (OR = 11.687, 95% CI: 5.279–25.876, *P* < 0.001), and minimum diameter of stent < 6 mm (OR = 2.047, 95% CI: 1.285–3.259, *P* = 0.003) (Table 2). These variables were included in the model fitting (Table 3). Besides, lesion type was also empirically considered strongly related to the poor outcome and was included in the machine learning models, although the *P* value of the OR is not statistically significant (OR = 0.653, 95% CI: 0.392–1.089, *P* = 0.102) (Table 2).

**Table 2:** Univariate logistic regression screening for candidate variables.

**Candidate variable**	**Univariate logistic analysis**	
	**OR (95% CI)**	***P*-value**
Age > 80 years	1.984 (1.153–3.412)	0.013
Hypertension	0.695 (0.420–1.149)	0.156
Diabetes mellitus	1.282 (0.818–2.008)	0.279
Hyperlipidemia	1.153 (0.706–1.883)	0.569
Myocardial infarction	0.862 (0.510–1.458)	0.580
Stroke	0.982 (0.544–1.774)	0.952
Current smoking	1.231 (0.734–2.064)	0.431
Rest pain or wound	1.658 (1.062–2.589)	0.026
ABI < 0.4	2.599 (1.633–4.137)	<0.001
Lesion type	0.653 (0.392–1.089)	0.102
Lesion location	1.648 (1.007–2.697)	0.047
Infra-popliteal runoff < 3	2.164 (1.371–3.417)	0.001
Target lesion length > 20 cm	3.143 (1.857–5.320)	<0.001
PACSS = 4	11.687 (5.279–25.876)	<0.001
Minimum diameter of stent < 6 mm	2.047 (1.285–3.259)	0.003

*OR, odds ratio; CI, confidential interval; ABI, ankle-brachial index; PACSS, peripheral artery calcification scoring system*.

**Table 3:** Variables for machine learning models.

**Variables**	**Index**		
Age	1: >80	0: ≤ 80	
Rest pain or wound	1: yes	0: no	
Ankle-brachial index	1: <0.4	0: ≥0.4	
Lesion type	1: CTO	2: stenosis	3: thrombosis
Lesion location	1: Iliac artery	2: femoropopliteal artery	3: mixed
Infra-popliteal runoff	1: ≤ 2 patent tibial arteries	0: 3 patent tibial arteries	
Target lesion length	1: >20 cm	0: ≤ 20 cm	
PACCS	1: grade 4	0: grade 0–3	
Minimum diameter of stent	1: <6 mm	0: ≥6 mm	

*PACSS, peripheral artery calcification scoring system; CTO, chronic total occlusion*.

### Machine Learning Model Training and Testing

**Figure 3:**
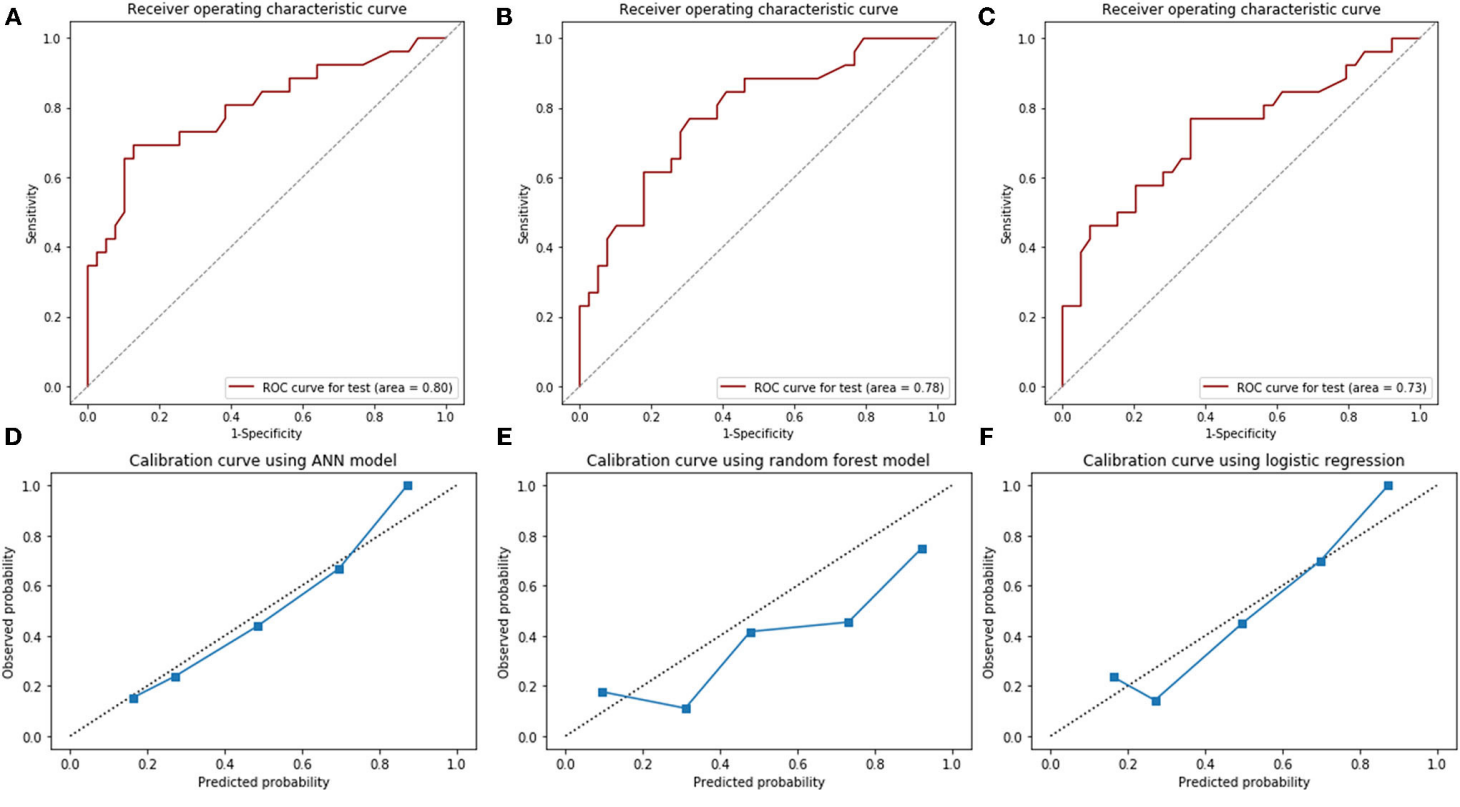
The receiver operating characteristic (ROC) curves (area under the curve) for artificial neural network model **(A)**, random forest model **(B)**, multivariate logistic regression **(C)**, and calibration curves for these models in the test set **(D–F)**. ANN, artificial neural network.

In the final ANN model, the structure was set as 1 input layer consisting of 9 variables, 1 hidden layer consisting of 12 neurons, and 1 output layer for binary classification, with dropout = 0.4 and 200 epochs according to the ten-fold validation process. Using this final ANN model, the ROCAUC was 0.80 (95% CI: 0.68–0.89) in the test set (Figure 3A), and the sensitivity and specificity at a cutoff value of 0.50 in the test set were 0.62 and 0.90, respectively (Table 4). Using the random forest model, The ROCAUC was 0.78 (95% CI: 0.66–0.87) (Figure 3B), and the sensitivity and specificity at a cutoff value of 0.50 were 0.73 and 0.72, respectively (Table 4). As a contrast, using the multivariate logistic regression, the ROCAUC was 0.73 (95% CI: 0.60–0.83) (Figure 3C), and the sensitivity and specificity at a cutoff value of 0.50 were 0.58 and 0.79, respectively (Table 4). The difference between the ROCAUC of the ANN model and the multivariate logistic regression model was 0.072 (95% CI: 0.017–0.127, *P* = 0.01). The difference between the ROCAUC of the random forest model and multivariate logistic regression model was 0.052 (95% CI: −0.036 to 0.140, *P* = 0.24). The ANN model and the multivariate logistic regression had good calibration performance, while the random forest model had poor performance (Hosmer–Lemeshow test, *P* = 0.73, *P* = 0.28, and *P* < 0.01, respectively, Table 4). The calibration curve of the ANN model depicting the observed and predicted probabilities was visually the closest to the perfectly calibrated line (Figures 3D–F).

**Table 4:** Comparison of performance between different models.

**Model**	**ROCAUC**	*P*-value^*^	Sensitivity^**^	Specificity^**^
Artificial neural network	0.80 (0.68–0.89)	0.01^a^	0.62	0.90
Random forest	0.78 (0.66–0.87)	0.24^b^	0.73	0.72
Multivariate logistic regression	0.73 (0.60–0.83)	–	0.58	0.79

*ROCAUC, area under receiver operating curve*.

* *DeLong test for significance of ROCAUC difference between two models*.

** *The sensitivity and specificity were calculated at probability cutoff value of 0.5*.

a *Comparison between artificial neural network and multivariate logistic regression*.

b *Comparison between random forest and multivariate logistic regression*.

**Figure 4:**
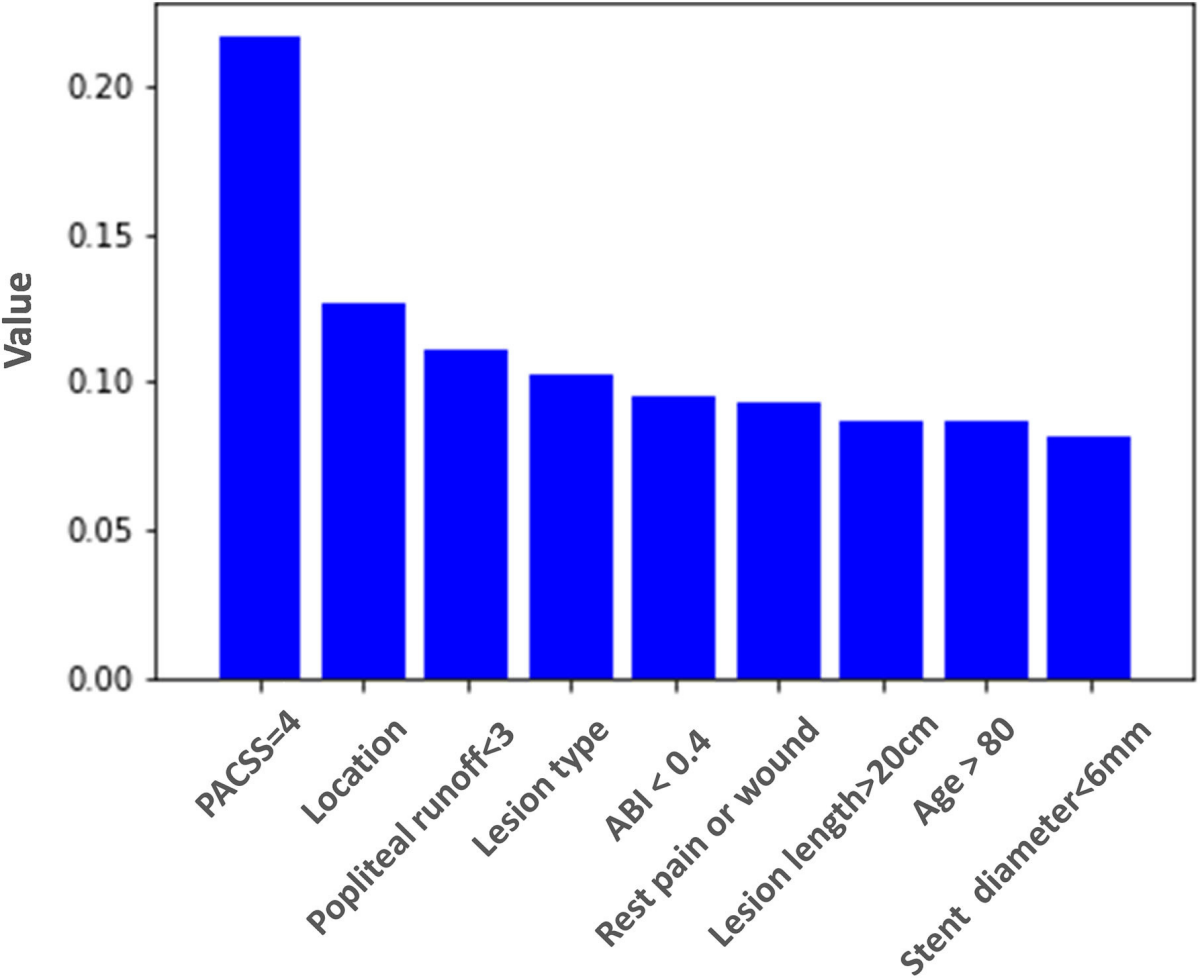
Importance ranking of variables used in the random forest model for prediction of 2-year major adverse limb event-free survival. PACSS, peripheral arterial calcium scoring system; ABI, ankle-brachial index.

The random forest model illustrated the importance ranking of the variables (Figure 4), indicating that the top three important variables were serious calcification (PACSS = 4), lesion location, and patent infrapopliteal runoff <3. The importance value of PACSS = 4 seemed much higher than any other variable and was considered as the key factor for prognosis.

## Discussion

### General Findings

In this study, we demonstrated both the ANN model and the random forest model outperformed the traditional multivariate logistic regression model to predict 2-year MFS after receiving PTA and stenting for lower limb ASO. Specifically, the ANN model showed significantly better discrimination than the multivariate logistic regression. Besides, the ANN model demonstrated good performance in terms of calibration rather than the random forest model. Accordingly, the modern machine learning models might make an accurate and stable prediction of prognosis in the treatment of lower limb ASO. Furthermore, machine learning could provide important ranking information of variables from the medical record, image, and intervention.

The classical risk stratification scoring systems, such as Fontaine or Rutherford grading system, mainly focus on the risk of limb amputation when receiving conservative therapy. The SVS WIFI scoring system extends the application range to evaluate the likelihood of benefit of revascularization. The TASC grading system classifies the anatomic factors of lesions into four grades and accordingly recommends the option of bypass or endovascular therapy (10). Recently, the GLASS has been proposed to evaluate the technical success and outcome of endovascular intervention for chronic limb-threatening ischemia (2, 11, 12). However, there were several limitations of the current scoring systems. First, one cannot calculate an exact probability of a certain prognosis for a single patient with these systems, and thus cannot make a precise prediction. Second, the correlation between the prognosis and a certain type of intervention such as PTA and stenting could not be assessed, since the intervention approach is not involved in these systems. Medical personnel such as vascular surgeon tends to evaluate the probability of a certain prognosis after a certain intervention and make the clinical decision in a more precise and personalized way. There is a demand for a prediction tool that could correlate prognosis with specific intervention patterns.

Nowadays, machine learning methods are increasingly applied in screening, identification, risk stratification, and prognosis prediction of circulatory system disease. Regression models, namely, penalized linear regression and logistic regression, and ensemble learning models, namely, bagging and boosting were reported to identify PAD and predict cardiovascular events or mortality. The random forest model, as a typical ensemble learning model, was proved to outperform other models in terms of identification of PAD with good discrimination and calibration in most pieces of literature (6, 7, 13, 14). However, there has been no report whether the random forest model could have a good predictive ability in the prognosis prediction after the intervention of PAD. The ANN model was characterized by strong non-linear classification and has been proved to have good sensitivity and specificity in the prediction of survival after repair for aortic disease, although the data size was not very big (15, 16). We supposed that the ANN model might also perform well in the field of PAD with the proper dataset. Thus, we decided to choose and compare these two typical models in this work.

Trained with the accumulation of complete clinical data, our machine learning-based models comprehensively considered systematic risk factors, lesion anatomic factors, and limb ischemia factors and make the precise prediction possible. Overall, both the ANN model and the random forest model had better performance than the traditional multivariate logistic regression. Considering the discrimination, the ANN model, but not the random forest model, prove a significantly higher ROCAUC than the multivariate logistic regression in the test, which meant the ANN model might have the better predictive ability in an unknown cohort. On the other hand, the ANN model showed lower sensitivity (0.62 vs. 0.73) and higher specificity (0.90 vs. 0.72) than the random forest model in the test set. We suppose that a higher specificity might contribute more to identifying MALE or death after PTA and stenting because these patients with a poor prognosis account for a minority. Besides, the ANN model also had good calibration performance, which meant that the calculated probability of the ANN model would be more reliable, and the real risk of a patient was less likely to be underestimated or overestimated. We supposed a well-trained ANN model might be clinically useful and help the vascular surgeon identify the candidate for PTA and stenting.

The variable importance ranking evaluation based on machine learning might also bring insights to clinical practice. The random forest model illustrated that serious calcification (PACSS = 4) of target lesion was the most critical prognostic factor. Several researchers have also indicated that PACSS was associated with primary patency loss, MALE, and even survival after endovascular therapy (17–19). With the result of machine learning, we figured out that serious calcification (PACSS = 4) seemed to be even far more important than any other variable. Some researchers indicated that the long bilateral calcification of lesions might restrict stent expansion and lead to negative remodeling of vessel lumen (13). Since all of the patients in our cohort received stenting treatment, we speculated that the factor of calcification might play a leading role in the in-stent patency loss and result in MALE. The random forest model also showed that factors of infrapopliteal runoff, target lesion location, and lesion-type ranked forefront. The age of the patient, the severity of limb ischemia, target lesion length, and minimal stent diameter seemed to have less impact on prognosis. These observations of variable importance ranking based on machine learning might help vascular surgeons consider priority and sequence of factors related to prognosis when making decisions of endovascular intervention.

## Limitations and Implementations

This study had several limitations. First, this study was based on a retrospective single-centered cohort and might generate selection bias and information bias. To reduce the biases, we utilized strict inclusion/exclusion criteria and organized two sophisticated staff groups to independently collect baseline information and follow-up data. Second, the machine learning-based models might still be underfitting, especially for the ANN model, because the sample size of the cohort was relatively small and some potential meaningful variables, such as the mechanical property of different stent types, have not been observed. Noteworthy, the predictive ability of the ANN model could not be improved by increasing layers in the pretraining phase (data not shown). The model training in the future should be performed on a larger cohort database. Third, the study focused on the patients who received stenting. However, a fair number of patients received PTA with or without debulking. A future machine learning model might involve this subpopulation and make the prediction more generalized.

## Conclusion

Machine learning could be applied to make an accurate prediction of 2-year MFS after PTA and stenting for lower limb ASO. The ANN and random forest models outperformed traditional multivariate logistic regression, in which the ANN model had better performance in discrimination and calibration. Machine learning-derived prediction tools might be clinically useful to automatically identify candidates for PTA and stenting and discriminate patients unsuitable for endovascular intervention and avoid inappropriate stent placement.

## Data Availability Statement

The raw data supporting the conclusions of this article will be made available by the authors, without undue reservation.

## Ethics Statement

The studies involving human participants were reviewed and approved by Ethics Committee of Zhongshan Hospital, Fudan University. The Ethics Committee waived the requirement of written informed consent for participation.

## Author Contributions

TP, XJ, WF, and ZD contributed to the conception and design. TP, XJ, HL, and YL contributed to the analysis and interpretation, data collection, writing the manuscript, and statistical analysis. WF and ZD contributed to the critical revision and overall responsibility. TP, XJ, HL, YL, WF, and ZD contributed to the final approval of the article. TP, WF, and ZD contributed to the obtained funding. All authors contributed to the article and approved the submitted version.

## Funding

This study was sponsored by the Shanghai Sailing Program (No. 20YF1406600), the National Natural Science Foundation of China (No. 82000452), the Project of Outstanding Academic Leaders of Shanghai Science and Technology Commission (No. 19XD1401200), and the Shanghai Engineering Technology Research Center for Interventional Therapy (No. 19DZ2250300).

## Conflict of Interest

The authors declare that the research was conducted in the absence of any commercial or financial relationships that could be construed as a potential conflict of interest.

## Publisher's Note

All claims expressed in this article are solely those of the authors and do not necessarily represent those of their affiliated organizations, or those of the publisher, the editors and the reviewers. Any product that may be evaluated in this article, or claim that may be made by its manufacturer, is not guaranteed or endorsed by the publisher.
